# Consumption

**Published:** 1859-07

**Authors:** 


### CONSUMPTION

Is called "Phthisis" in scientific books, pronounced by some in this country as if written Teesis. We heard some of the Napoleons of medicine in England, some fifteen or twenty years ago, call it Thysis. Now if "Thysis" is Phthisis, and Phthisis is consumption, it may be well to know clearly what is the nature of it, inasmuch as about one person out of every six is destined to die of it.

A French writer in December, 1858, defined it thus :—

"Phthisis is a diathesis, depending on the want or undue waste of the oxydisable phosphorus normally existing in the animal economy."

Another says, "phthisis is the oxydation of the exudation corpuscle."

These definitions are perfectly plain to those who made them—perhaps.

The latter means, that in consumption, the blood is squeezed or exudes through the coats of the blood vessels, then turns into a small, hard, roundish body, which is called a tubercle; if this tubercle is formed in the lungs, it ends in consumption; if in the joints, it becomes white swelling, thus giving a name to the disease which it occasions, according to the locality. As to the lungs, the tubercle begins to soften, that is, it is oxydized, is burnt up, and as the tubercle is supplied by the blood, the essential parts of the blood are used up, burned, and the man has no blood scarcely; he looks as pale as a sheet, and with the failure of blood, there is a failure of strength, and flesh, and life, and with the tubercle, the lungs themselves are consumed.

The other definition is more resplendent still, and the practical conclusion resplendenter. It is, that consumption is a disease of the whole man, which is very true, as it includes soul, body, and estate. The mind is diseased, because the consumptive is usually the very last person to be convinced that he has the malady, coming to his senses however a few days before death. That the whole of the body is diseased, is wasting away, is painfully evident to all; and that the estate is also wasting, very many have a feeling sense of the truth of this same, for it requires "considerable" money to purchase everything that every body advises, the almost invariable inducements thereto being, "it can do no harm, if it does no good, and it cured Mr. Smith, who was a great deal worse off than you, and he would have been living yet if he hadn't died so soon."

The Frenchman goes on to say, that not only is the whole man diseased in consumption, but the disease is owing to the fact, that either the man has not enough phosphorus in him, or it burns up too fast. A general observer might reply, "I'm not so certain about that, for there could be no bones without phosphorus, and a consumptive has more bone than any thing else; in fact he is too bony; so full of bones sometimes, that the skin is worn through in holding them." Hence an English great name says, it is a mistake, for the phosphorus in a man, instead of burning up too fast, won't burn at all. So that John Bull, has the better of "boney" this time.

The Frenchman, like old Rough and Ready, not knowing himself beaten, goes on to say, that consumption being the undue waste or want of phosphorus, the cure of it consists in giving the body more phosphorus. That being the case, all that a consumptive has to do, is to turn politician, and swallow every "*loco foco*" he comes across. There's hope for whiggery yet, for they have only to go to Florida, and the "sunny south" any December, and enlist all the consumptives, and just turn them loose among the thickest of the "unterrified."

But to use a plainer form of speech, the *loco foco* above spoken of, is a "match," the better kind of match, made of phosphorus, not having the objectional brimstone odor. This form of phosphorus, however, the Frenchman does not advise; for as bones contain phosphorus, the idea is not to swallow *loco focos*, but to burn up the bones of the dead, powder them, and swallow them down thrice a day, as much as may be taken upon a dime.

After the Frenchman printed his book, druggists scattered themselves all over the country, to gather up the bones of the dead, whether of cat, dog, or human, is of no consequence, for bone is bone, always and everywhere; for if bone be not bone, what is bone? It must be something else, and consequently is not bone at all. There must be some mistake about it somewhere, for the Frenchman has failed to cure his consumptives, either because the real, identical, original bone was not given; or bone, the "hypophosphite" is no remedy. As we were saying, on the publication of the first edition of M. Churchill's book, its main position was denied, and before he issued a second edition, he concluded to wait until he could be "able to settle the question of the existence or non-existence in the human body, of phosphorus in a burnable condition." Reasonable people would have thought that it would have been better to have settled that question first. He thinks he will succeed. Suppose he does succeed in proving that consumptives need phosphorus, that will not prove that an artificial supply of it will cure consumption. All disease consists in a defect of some element. Medical research has found out, in many cases, what that element is. Many for example need iron ; a forlorn few need brass ; but of the millions who have failed to be benefited by taking iron rust, finely powdered, any eminent physician may testify.

Every element of the human body is found in the food which nature prepares for man ; our food is the vehicle through which that element is supplied, and that is the natural mode of supply. Thus it is, that in many remembered cases, sick persons became possessed of an appetite for a particular kind of food, and on eating it got well, because, as it were, nature knew that the body needed the element which that kind of food most largely abounded in. Very beautiful instances of this are recorded in medical works ; see the back volumes of *Hall's Journal of Health*, under the head of "Instinct."

As then, all admit that consumption is a general disease, involving the whole body, it would seem that every element was needed, and that to get a full supply of every element in a natural way, in a safe way, and in due proportion, the best plan is to get up a good appetite and a good digestion ; then eat, and drink, and breathe the best of every thing, meat, bread, water, air. That this has proved happily efficient in multitudes of cases, there is various proof, strong as of any fact in nature, but the folly of man is such, that in nine cases out of ten, he will "take" any thing else, every thing else, how horrible soever it may be, sooner than meat, and bread, and water, and air.

The ignorance or the perversity of the human mind is such, that in consumptive diseases, the intelligent physician has to ransack all nature to find arguments to induce his patient to expose himself sufficiently to out door air. In nine cases out of ten, there is such an insane dread of "taking cold," that the patient hugs the stove, and—death at the same time, or hurries off to a southern climate, where the atmosphere is loaded with dampness, and the more deadly miasm, the emanations from rotting leaves and wood which cover every inch of soil, instead of going where the air is too cold for decay or for holding moisture, hence must be the purest, and most nutritious in all nature.

There must be something in consumptive disease which infatuates the mind with error, or warps it by delusions. It would seem that any man at a small remove from idiocy, might know that pure air was the best for breathing, in all forms of sickness; and yet within two or three years, a large number of newspaper editors lent themselves to the advocacy of "medicated inhalation" for the cure of consumption, and other diseases of the lungs and throat; the convincing argument on the part of the *Herald*, *Tribune*, and *Times*, being advertisements, at the rate of two or three hundred dollars a piece. Against printing a mere advertisement, we say nothing; but we do reprobate the recklessness and the folly of any editor who lends himself to be the tool of design or ignorance. Even so staid and respectable a paper as the *Commercial Advertiser*, lent itself to the propagation of the delusion by certificates and otherwise, of the wonderful efficacy of medicated inhalation in diseases of the throat and lungs; the result was, to send their confiding readers in crowds, to breathe impure air, at the rate of twenty-five dollars a month for the privilege. Day after day, and week after week, the city was flooded with advertisements in the penny press, five columns long, in laudations of the treatment, until "every body went there for every thing;" and finally, as we personally know, some men were set to breathing medicated air for diarrhœa and dysentery. And what is the history of to-day? The Boanerges of inhalation has long since left the city. The very name of "medicated inhalation" had become such a stench in the nostrils of the people, that for sometime previous to his departure, the silver sign had been removed from the door, and the occasional advertisement of five lines long, instead of five columns, never mentioned the once cabalistic words.

Let it be remembered in conclusion, that "consumption," in the usual acceptation of the term, is a destruction of the substance of the lungs, and in proportion as they are destroyed, is less air consumed, hence the great need, that what air is breathed, should be the purest possible; just as if a man has less food given him than the system needs, the most certain plan of protracting life, is for him to arrange that the food which he does consume, should be of the most nutritious kind that can be had.

